# Intentional practice: a common language, approach and set of methods to design, adapt and implement contextualised wellbeing solutions

**DOI:** 10.3389/frhs.2023.963029

**Published:** 2023-06-16

**Authors:** Ivan J. Raymond

**Affiliations:** Clinical, Research and Education Director, LBI Foundation, Adelaide, SA, Australia

**Keywords:** intentional practice, intervention design and implementation, contextualisation, knowledge translation, implementation science, complexity, trauma-informed practice, evidence-based practices

## Abstract

Reducing the “science-to-practice” gap has gained significant attention across multi-disciplinary settings, including school psychology and student wellbeing, trauma-informed practice, community and human services, and clinically focused health care. There has been increasing calls for complexity and contextualisation to be integrated within the implementation science literature. This includes the design and implementation of interventions spanning “systems” (whole-of-community capacity building initiatives), “programs” (e.g., evidence-based programs, clinical interventions) and “moment-to-moment” support or care. The latter includes responses and communication designed to deliver specific learning, growth or wellbeing outcomes, as personalised to an individual's presenting needs and context (e.g., trauma-informed practice). Collectively, this paper refers to these interventions as “wellbeing solutions”. While the implementation science literature offers a range of theories, models and approaches to reduce the science-to-practice gap in wellbeing solution design and implementation, they do not operationalise interventions into the “moment”, in a manner that honours both complexity and contextualisation. Furthermore, the literature's language and content is largely targeted towards scientific or professional audiences. This paper makes the argument that both best-practice science, and the frameworks that underpin their implementation, need to be “sticky”, practical and visible for both scientific and non-scientific knowledge users. In response to these points, this paper introduces “intentional practice” as a common language, approach and set of methods, founded upon non-scientific language, to guide the design, adaptation and implementation of both simple and complex wellbeing solutions. It offers a bridge between scientists and knowledge users in the translation, refinement and contextualisation of interventions designed to deliver clinical, wellbeing, growth, therapeutic and behavioural outcomes. A definitional, contextual and applied overview of intentional practice is provided, including its purported application across educational, wellbeing, cross-cultural, clinical, therapeutic, programmatic and community capacity building contexts.

## 1. Introduction

There is wide acceptance that evidence-based practices deliver stronger consumer outcomes and optimise the delivery of finite health, care, wellbeing and clinical resources. Implementation science has developed as a multi-disciplinary body of literature to reduce the “science-to-practice” gap in the delivery of clinical, trauma-informed and growth-focused services across health, care, therapeutic, education and community-based settings (1–4). The literature has evolved to identify scientific methods, models and processes that intentionally seek to strengthen intervention delivery and outcomes (5).

Despite increasing multi-disciplinary interest, the accumulation of scientific knowledge within the implementation science literature remains slow, with few new insights in the last decade (4). An overarching intent of this paper is to both introduce and inspire novel insights that tap the interface between theory and application within real-world contexts, a key area of development for the field (5). The paper introduces “intentional practice” as a common language, approach and set of methods that is purported to support the design, adaptation and implementation of both simple and complex wellbeing solutions (from the “system” to the “moment”). By definition, a “wellbeing solution” is any strategy, intervention, program or response that is designed to deliver a wellbeing, growth, learning, developmental, behavioural or therapeutic outcome. Intentional practice is designed to bring together scientific and non-scientific audiences around a “shared intent” in wellbeing solution design and implementation. This responds to a call within the implementation science literature to embrace complexity and contextualisation (6, 7), and the important role of participatory processes to aide this outcome (8, 9).

To set the context for introducing intentional practice, the paper will define wellbeing solutions, and then introduce the language of “stickiness” as a non-scientifically worded construct to operationalise the desired outcome of optimal wellbeing solution design and implementation. The paper will then detail the heterogenous nature of wellbeing solutions across educational, trauma-informed, community services and clinically focused health care, and articulate key considerations for implementation scientists across these disciplines. Intentional practice is then introduced as a design and planning process to respond to gaps in existing implementation science models, including: (1) the heterogenous nature of wellbeing solutions (“system” to the “moment”), (2) complexity and contextualisation, and (3) engaging all knowledge users in implementation science constructs and approaches.

## 2. What are wellbeing solutions?

Intentional practice was first conceptualised as a method to guide caregivers, teachers, practitioners, researchers and program developers to deliver growth outcomes, and reduce unintentional harm, through the design and implementation of clinical and non-clinical interventions. This intent remains current, however the term “intervention” which was prevalent in early writings (10–12) has been replaced with “wellbeing solution”. The language of intervention was found to be a key barrier to accessibility, notably within the Australian context where the term is associated with the suppression of Aboriginal people through government-led “interventions”.

By definition, a wellbeing solution is any strategy, intervention, program or response that is designed to deliver a wellbeing, growth, learning, developmental, behavioural or therapeutic outcome. In other words, intentional practice supports the design, adaptation and implementation of interventions or responses across multi-disciplinary settings; both clinical and non-clinical. Table 1 summarises the heterogenous and multi-levelled nature of wellbeing solutions in which intentional practice is purported to offer utility, and the diversity of knowledge users who are potentially engaged in their design, adaptation and implementation. It is possible intentional practice offers value beyond these applications, but the paper limits itself to this restricted focus.

**Table 1:** Wellbeing solutions and associated knowledge users.

Setting	Wellbeing solutions	Knowledge users
Trauma-informed practice	• Milieu-based program model • Practice framework • Trauma-focused interventions • Behaviour, growth, care and safety planning • Intentional coaching and restorative practice conversations • Moment-to-moment trauma-informed practice	Leaders, program developers, psychologists, practitioners, residential care workers, support workers, foster carers, educators, clients
School psychology and wellbeing	• Whole-of-school wellbeing approach • Psychological and developmentally responsive interventions • Social-emotional learning (SEL) programs and wellbeing curriculum • Positive psychology interventions (PPIs) • Positive behavioural support • Behaviour, growth and safety planning • Intentional coaching and restorative practice conversations • Moment-to-moment trauma-informed practice	Principals, site leaders, educators, administration staff, school psychologists and counsellors, wellbeing leaders, families, students
Community and human services	• Practice framework (therapeutic or trauma-informed) • Evidence-based interventions individualised to local need/context • Case management and planning • Behaviour, growth and safety planning • Intentional coaching and reflective conversations • Moment-to-moment trauma-informed practice	Policy makers, program developers, agency leaders, social workers, youth workers, support workers, foster carers, families, clients, allied health practitioners
Clinically focused health care	• Clinical practice framework • Clinical interventions • Psychological interventions • Case formulation, management and planning • Care planning • Moment-to-moment trauma-informed practice	Doctors, nursing staff, clinicians, allied health practitioners, leaders, policy makers, administration staff, non-clinical support workers, clients, families

## 3. Contextual foundations of intentional practice

Intentional practice has been refined through a participatory process, spanning 10 years, involving numerous scholarly and applied collaborations. The conceptualisation and refinement has been strongly grounded in the knowledge translation and implementation science literatures, and the deconstruction of scientific evidence for non-scientific audiences (e.g., 13).

### 3.1. Knowledge translation and implementation science

Knowledge translation can be defined as the exchange of scientific evidence across relationships and systems, which involves a process of synthesis, dissemination and application of best-practice knowledge (4, 9, 14). Straus et al. argue this should be both targeted and intentional; supported through an iterative process between knowledge creation and its implementation. Knowledge translation includes the co-production of knowledge and its strategic communication through resourced and intentional strategies (15).

In contrast, implementation science brings focus to “how to implement” an intervention or scientific knowledge, and it represents the “to” in science-to-practice (16). It articulates scientific methods that promote the uptake of evidence-based approaches into service delivery and care, as operationalised through organisational, structural, financial and professional strategies (17). The implementation literature is made practical through a range of models, theories and approaches (see 5), and best practice summary guidelines (e.g., 18).

### 3.2. “Stickiness” as a desired outcome of knowledge translation

The desired outcome of implementation science and knowledge translation is that evidence-based constructs “stick” in practice. In other words, scientifically grounded processes and methods become “sticky” in the minds, decision making and actions of individual and collective knowledge users. The construct of stickiness is drawn from the positive education literature. White (19) discusses the reasons and rationale for why positive psychology content and processes (e.g., science of wellbeing, positive psychology interventions) did not translate nor “stick” across a whole-of-school wellbeing initiative. Stickiness represents a cognitive framing element or non-scientific cue that can be applied by all knowledge users to identify the desired outcome of implementation and knowledge translation. The knowledge translation literature highlights the key role of “mental short-cuts” and framing elements to support the uptake and application of scientific-based constructs (13). The iterative development of intentional practice has been motivated by implementation science methods and processes becoming sticky in the decision making processes of diverse knowledge users.

### 3.3. Multi-disciplinary and multi-levelled applications

The conceptualisation of intentional practice has been informed by implementation science applications across multi-disciplinary domains, including trauma-informed practice, school psychology and wellbeing, community and human services, and clinically focused health care. This section reviews these domains and summarises key underpinning themes.

#### 3.3.1. Trauma-informed practice

Trauma is an event or experience that overwhelms an individual's coping ability; often associated with the absence of emotional and/or physical safety. Trauma-informed practice has emerged as a set of best-practice principles that are brought to focus in the care, support and teaching of people with trauma backgrounds (20). These principles include safety, trust, choice, collaboration and empowerment (20, 21), and they are designed to be operationalised to individual and collective context. Context is a key consideration in trauma-based intervention planning. To illustrate this point, the impact of childhood abuse and trauma is strongly mediated by a child's developmental age (22). Trauma interventions should therefore be responsive to a child's individual developmental needs and context (23, 24).

Trauma-informed practices are being increasingly embedded across human and community services (20), education (25), and residential care and in-patient treatment settings (21). Improving implementation quality has been highlighted across the literature (26). At the core of trauma-informed practice is the role of “deeply personal, human relationships” (27, p. 97), which support both growth and healing (28). Evidence-based trauma interventions for children and young people focus on the rebuilding of healthy attachment and self-regulation capacity. This is delivered by caregivers, teachers and clinicians through individualised moment-to-moment support, care and coaching (29). The focus of intervention is to respond to the child's unique needs and context in the moment of support. To demonstrate, consider a child presenting with elevated emotions. The supporting adult is required to develop a personalised response or intervention that responds to the child's specific needs and context, in that moment of time (30). In short, trauma-informed practice can be operationalised as thousands of micro contextualised interventions (or learning or teaching moments) that collectively deliver larger self-regulation and attachment outcomes.

As illustrated in Table 1, trauma-informed practice can be operationalised through a variety of wellbeing solutions, including: whole-of-site (or agency, school, program) to moment-to-moment support, care and teaching. Both clinical and non-clinical knowledge users have key roles in the design and implementation of trauma-informed care. The trauma science themes of contextualisation, diversity of knowledge users, operationalising wellbeing solutions into the moment, and psychological safety were foundational considerations in the early conceptualisation of intentional practice.

#### 3.3.2. School psychology and wellbeing

Schools and education providers deliver a range of strategies, programs and responses that meet broader student developmental and growth needs (e.g., trauma, disability, wellbeing, mental health). This is captured within diverse literatures such as school psychology, positive education, trauma-informed education and school connectedness. Implementation science remains within its infancy across the school psychology literature, with increased literacy and application warranted (2, 31). In a recent review, Shoesmith, Hall (32) conducted a review of the facilitators and barriers of implementing health behaviour interventions within an educational setting. In this study, supported by others (33), local school-based contextual factors were identified as having a key role to explain intervention stickiness.

Schools represent complex eco-systems, and evidence-based practices can be applied at multiple layers and by diverse knowledge users (see Table 1). This includes through the implementation of whole-of-school approaches to wellbeing, specific strategies and principles to respond to cohorts of students (e.g., trauma, disability), evidence-based psychological and developmental interventions, social-emotional learning programs, and individualised student behaviour management or growth planning. The school-based themes of contextualisation, multi-levelled wellbeing solutions and diverse knowledge users were key considerations in the conceptualisation of intentional practice.

#### 3.3.3. Community and human services

Community services deliver support, care, case management and interventions by a diversity of skilled and semi-skilled personnel (see Table 1). Across these settings, “the practitioner is the intervention” (34, p. 532), where scientific constructs require embedding into systems and service delivery (35). This systemic implementation is often facilitated through practice frameworks or logic models (36, 37), with purveyors and third party experts having a key role to guide and support systemic implementation (34, 38). As seen in Table 1, community and human service settings also include a number of nested interventions. These include evidence-based packages or programs, trauma-informed practice, and individualised case planning which are nested within broader practice or therapeutic frameworks. The themes of the “practitioner as the intervention” and nested interventions were highly influential in the early conceptualisation of intentional practice.

#### 3.3.4. Clinically focused health care

The role of implementation science is well established within the health care literature. This includes the delivery of evidence-based practices across general practice, in-patient and out-patient services, psychology and psychiatry, and allied health (3, 4). The breadth of evidence-based interventions and knowledge users is summarised in Table 1. Health care has become significantly complex, where contextualisation remains at the forefront of service delivery (39). Across the psychological literature there are frameworks and methods to navigate complexity and contextualisation. For example, cognitive-behavioural therapy (CBT), an evidence-based clinical intervention, is designed to be adapted and implemented through an individualised “case formulation process” (40). Le, Eschliman (41) describes a current feature of mental health care is “task sharing”. This is where non-clinicians (e.g., support workers, case managers) are being asked to be active in the planning and implementation of mental health and wellbeing services. The operationalisation of task-sharing was an early design feature of intentional practice.

#### 3.3.5. Summary and application to current implementation models

Three key themes are brought forward from the previous sections. These are reviewed against the degree current implementation science models respond to key features of the theme. Summary reviews of implementation models are not offered in this section, but are available elsewhere, specific to generalised applications (5), as well as therapeutic residential care (26) and child welfare and mental health services (42).

An overarching theme is that complexity and contextualisation are foundational considerations in the design, adaptation and implementation of wellbeing solutions. While traditional implementation models have been founded upon a pipeline or linear model (6), there has been increasing scholarly interest to embrace complexity in the design, operationalisation and implementation of interventions (2, 6, 7, 43–46). Intentional practice responds to this call.

A second theme is the heterogenous and multi-levelled spectrum of wellbeing solutions. Evidence-based wellbeing solutions are often embedded or nested within broader interventions, and intervention design and implementation can be operationalised into moment-to-moment processes. This includes the building of emotional and psychological safety in the moment of care (e.g., trauma-informed practice). Existing implementation models and theories do not guide intervention planning within moment-to-moment support, where interventions are contextualised to changing support needs and presentation. This remains a unique feature of intentional practice.

The final theme is that knowledge users are diverse, and both scientific and non-scientific voices (see Table 1) are often required to come together in wellbeing solution design and implementation. Traditional implementation models and theories are largely operationalised through scientific or professional language and methods, with the constructs not tailored to non-scientists (e.g., teachers, caregivers, support workers). Implementation strategies, frameworks and models are not routinely visible to all knowledge users. Drawing upon the words of Nilsen (5): “theorizing about implementation should therefore not be an abstract academic exercise unconnected to the real world of implementation practice”. This paper makes the case that implementation science constructs should be tailored, visible and sticky for all knowledge users. This remains a key motivational design feature of intentional practice.

## 4. Introducing the language, approach and methods of intentional practice

In this section intentional practice is introduced to guide the planning and implementation of wellbeing solutions, in a manner that upholds:

• The heterogenous nature of wellbeing solutions (“system” to the “moment”).
• Complexity and contextualisation.
• Engaging all knowledge users in implementation science and knowledge translation constructs and approaches.

### 4.1. Positioning definition

To date, intentional practice has not been coherently and consistently defined within the literature. Earlier scholarly writings brought a definitional focus to its “application” (10–12), which did not provide guidance in how intentional practice was positioned within the broader scientific literature. This impacted on its translational capacity. Today intentional practice has consolidated its definition as follows:

*“intentional practice is a common mindfulness-based language, approach and set of methods to design, adapt and implement safe and high impact wellbeing solutions from the system to the moment”*.

This positioning definition is systematically explored.

### 4.2. Wellbeing solutions from the “system” to the “moment”

**Figure 1:**
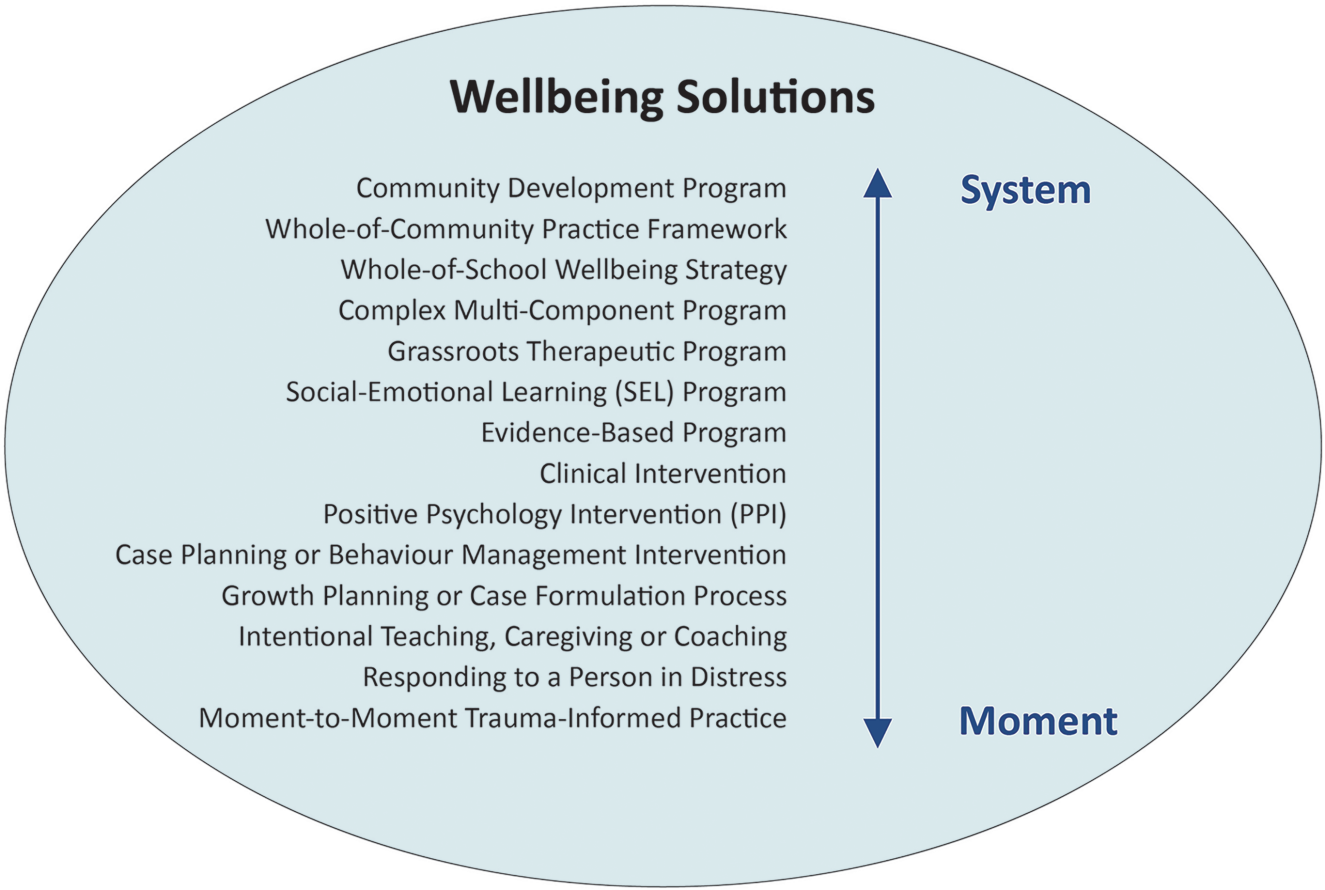
Examples of wellbeing solutions spanning the “system” to the “moment”.

Intentional practice categorises wellbeing solutions as spanning the “system” to the “moment” (see Figure 1). The term “system” is applied to denote a wellbeing solution that brings focus to multiple people or at a community or collective level. System-based wellbeing solutions include whole-of-school approaches and community capacity building initiatives. In contrast, the term “moment” refers to a wellbeing solution or response that is delivered through interactions between two or more people. For example, this may include a parent providing support to a child in distress, or a coaching or counselling intervention. Intentional practice upholds the potential of moment-to-moment interactions or support between two humans, even if it is only brief, to have a powerful effect (positive or negative) on human wellbeing and functioning.

### 4.3. Safe and higher impact wellbeing solutions

The early conceptualisation of intentional practice was informed by the implementation science literature. This included a scholarly focus to isolating intervention features (or covariates) associated with stronger effect sizes. Following a review of cross-disciplinary meta-analyses (e.g., 18, 47, 48), Raymond (11) proposed five principles associated with interventions delivering stronger social, emotional and psychological outcomes. These principles underpinned the conceptualisation of intentional practice and key foundational models. In response, intentional practice has been designed to support the delivery of “higher impact” wellbeing solutions, or the delivery of simple and complex wellbeing solutions associated stronger outcomes or effect sizes.

Following conceptualisation, early testing and refinement occurred across child welfare settings, with intentional practice guiding the responses of adults and programs supporting children with complex trauma backgrounds. The trauma-informed literature was highly significant in this developmental process in two main ways. First, it uplifted the role and importance of emotional and psychological “safety” within the design, adaptation and implementation of wellbeing solutions (or contextualised interventions). Second, it brought focus to the importance of the “moment” or singular interactions, between two or more people, as being meaningful interventions in their own right (as previously discussed).

### 4.4. Intentional practice as a language

Intentional practice has been designed to offer a common language to support and guide multiple knowledge users to design, adapt and implement wellbeing solutions. Table 2 provides a glossary of foundational language and definitions which are designed to be sticky in the minds of knowledge users. These definitions are further operationalised in subsequent sections.

**Table 2:** Foundational language and definitions of intentional practice.

Construct	Definition
Intentional practice (in application)	The bringing of mindful awareness to the “what” and the “how” within the design, adaptation and implementation of the wellbeing solution.
Wellbeing solution	Any strategy, intervention, program or response that brings an intent to deliver a wellbeing, growth, learning, developmental, behavioural or therapeutic outcome.
System	A wellbeing solution that spans an entire community, school or agency, and includes multi-levelled or nested wellbeing solutions.
Moment	A wellbeing solution or response that is delivered or operationalised through the interactions between two or more people.
Approach (principles and lens)	The worldview, mindset and energy brought to the design, adaptation and implementation of wellbeing solutions.
Mindful awareness	Open, curious and non-judgemental lens that is brought to the design, adaptation and implementation of wellbeing solutions.
Intent (“What”)	The purpose, aim or desired outcome of the wellbeing solution.
Components (“How”)	The actions, strategies, scripts, language and programmatic features that are designed to deliver the intent of the wellbeing solution.
Growth intent	A person, program or community brings an intent and energy to “growth”, or building the capacity of individuals and communities for improved whole-of-life outcomes.
Shared intent	A community or group has a shared and co-created awareness of: (1) what is happening in the wellbeing solution context, (2) what is the intent or desired outcomes of the wellbeing solution and (3) how this will be collectively actioned.
Building blocks	Smaller domains of growth competencies or intent that are operationalised through the language of: (1) awareness, (2) skills and (3) mindsets, and are aligned to larger growth outcomes or goals.
Set of methods	The way in which the intentional practice approach is applied, operationalised or used across different contexts or situations. This includes through models, process steps and critical questions.
Awareness	Knowledge and insight about self, others, world and future.
Skills	Actions that take knowledge into action through coping responses and behaviours.
Mindsets	The attitude or beliefs related to how someone sees themselves, others, their capacity, their world and future.
Critical questions	Questions that are designed to “ground” the design, adaptation and implementation of the wellbeing solution, and bring people and communities back to critical intentional practice design principles and planning features.
Life Buoyancy Model	A foundational model of intentional practice. It is operationalised as a logic model of short-, medium- and long-term outcomes (“what”), and associated intervention or wellbeing solution components (“how”).

### 4.5. Intentional practice as an approach

In early writings, intentional practice was described as both an “approach” (11) and “methodology” (10, 12, 30, 49), with the terms conflated. Intentional practice is now defined as both, with each term having a distinct meaning. The term “approach” can be described as the way individuals, programs or communities approach the design, adaptation and implementation of wellbeing solutions through worldview, mindset and energy. It represents the direction, angle or lens that is brought to wellbeing solution formulation and delivery. In contrast, “methodology” operationalises the process or the way in which wellbeing solutions are formulated through modelling, critical questions and process-based steps.

**Figure 2:**
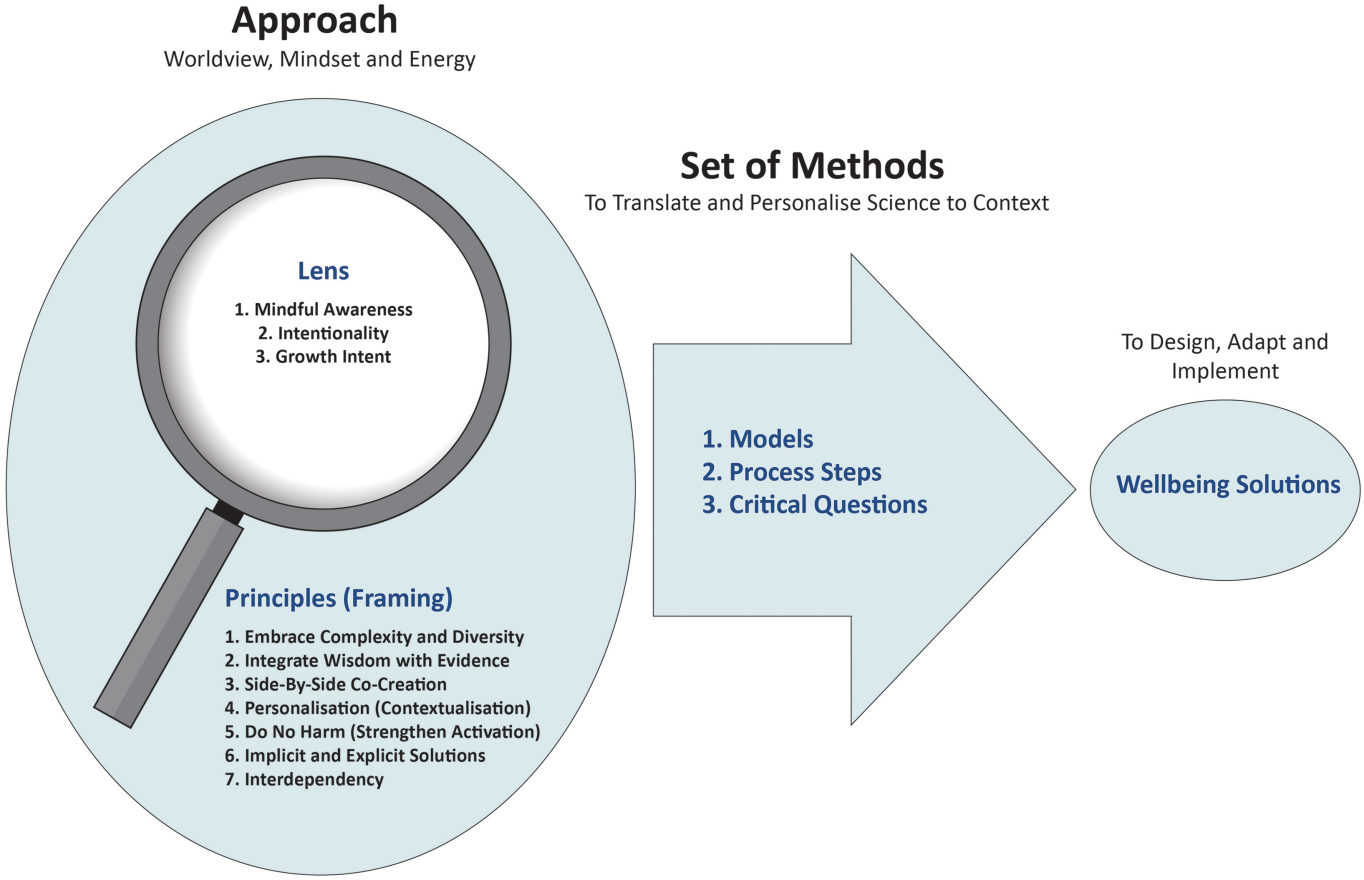
Intentional practice as an approach and set of methods to design, adapt and implement wellbeing solutions.

As an approach, intentional practice is analogous to the magnifying glass (see Figure 2). A magnifying glass has both a frame and a lens. The frame is represented by seven key principles that “frame” intentional practice as an approach. In other words, individuals, programs and systems “hold onto” these principles in the design, adaptation and implementation of wellbeing solutions or contextualised interventions. These principles are named and defined in Table 3.

**Table 3:** Principles underpinning and framing intentional practice.

Key principles (frame)	Definition
Embrace complexity and diversity	Wellbeing solutions should embrace complexity, human diversity and multiple worldviews, and move away from one-size-fits-all or reductionism.
Integrate wisdom and evidence	Wellbeing solutions should uplift and value both local wisdom (individual and community voice) with external world evidence (best-practice science, external sources).
Side-by-side co-creation	Wellbeing solutions should be co-designed and co-created with multiple voices positioned in a side-by-side manner or with equal power relationships, including the intended audience of the wellbeing solution.
Personalisation (contextualisation)	Wellbeing solutions should be intentionally personalised or tailored to the unique context and needs of individual and community; drawing upon an ecological, systems or broad-based understanding of human functioning.
Do no harm (strengthen activation)	Wellbeing solutions should seek to minimise unintentional “harm”. The potential impact of wellbeing solutions can range on a continuum from activating (building capacity for thriving outcomes) to suppressing (causing harm). The one wellbeing solution has the potential to be experienced in both ways, and strengthening the activating properties of wellbeing solutions remains paramount.
Implicit and explicit solutions	Wellbeing solutions should draw upon a range of intentionally delivered strategies and responses drawn from both implicit (e.g., indirect learning processes, role modelling) and explicit learning approaches (e.g., training, coaching).
Interdependency	Wellbeing solutions should express a commitment to a symbiotic or interdependent relationship between people, their community, nature and the broader systems around them.

The principles are written in language that is designed to be understood and applied by non-scientific audiences. They align to the epistemological, political and ethical assumptions of Systems Informed Positive Psychology (SIPP; see Table 1 in 45). Furthermore, at the heart of both intentional practice and SIPP is “interdependency”, or the co-existent or symbiotic relationship between humans, community, environment and wider systems. This reflects the call for increased integration of the complexity and implementation science literatures (6, 7).

As symbolised in Figure 2, intentional practice as an approach can also be represented as a “lens”. This captures the worldview, mindset and energy individuals, programs and systems constantly hold in their mind through the design, adaptation and implementation of wellbeing solutions. There are three key features of this lens.

#### 4.5.1. Mindfulness (mindful awareness)

The central lens of intentional practice is mindfulness, or mindful awareness. For this reason, intentional practice is defined as a “mindfulness-based approach”. Mindfulness represents an open, non-judgemental and curious awareness to any experience or event (50, 51); underpinned by focused attention (52). Mindfulness upholds a flexible and adaptive processing of information (53), and being willing to shift and change one's worldview or frame of reference. Within intentional practice, the features of openness, non-judgemental awareness and curiosity are continually brought to focus in the design, adaptation and implementation of wellbeing solutions.

#### 4.5.2. Intentionality

“Intentionality” can be understood as a unifying term that offers a “cognitive shortcut(s) that nonscientists bring to the discussion of complex issues” (13, p. 30). The term “intent” describes the purpose and aim associated with a wellbeing solution, including its desired outcomes (“what”). In application, intentional practice can be defined or operationalised as the bringing of “mindful awareness to the “what” and the “how” (e.g., strategies, responses, methods) within the design, adaptation and implementation of the wellbeing solution”. This applied definition is further operationalised through the following key questions:

• What is the intent, energy or philosophy driving the wellbeing solution (or strategy, response, program, intervention)?
• What outcome is at the focus of the wellbeing solution (or intervention)?
• How, or by which method or process, is this outcome being achieved?

#### 4.5.3. Growth focus (or growth intent)

The question **“**what is the intent, energy or philosophy driving the wellbeing solution?” is foundational within intentional practice, and ensuring that wellbeing solutions have increased potential to have an activating (or growth effect), as opposed to causing unintentional harm (suppressing in nature). The reason intentional practice explicitly uplifts “growth” as an intent is that many wellbeing solutions or interventions are embedded within agencies and systems that have competing needs and different intents. For example, if we consider the implementation of an evidence-based social-emotional program delivered by educators for students with self-regulation problems. The intent educators bring to the implementation of the intervention could be to: (1) “change student behaviour”, (2) “manage student behaviour and risk impact on other students”, (3) “fix the problem”, (4) “punish or consequence student behaviour” or (5) “grow student self-regulation capacity”. Each of these underlined categories brings a different intent, energy and focus to the implementation of the wellbeing solution. By naming “growth” as an intent, strength focused practice philosophies, as aligned to positive psychology (54), are both magnified and illuminated in the implementation (10). This point highlights the importance of implementation strategies bringing “alignment” between the intervention and the characteristics of an organisation, agency or community (55), including prevailing community attitudes and values (33, 56).

In short, intentional practice uplifts a growth-focused lens to the design, adaptation and implementation of wellbeing solutions. The intent or purpose of interventions are to “grow” (or build) the capacity of individuals and communities for improved whole-of-life outcomes (11). This growth-focused orientation is operationalised as individuals and programs adopting a “growth intent”. The construct of growth intent has been drawn from the positive psychology literature on optimal functioning (54), self-determination (57) and growth mindset (58). In intentional practice, this can be made practical through the critical question: “what is the growth intent of my support, this program or wellbeing solution?”.

### 4.6. Intentional practice as a set of methods

Intentional practice as a “set of methods” (or methodology) represents the way the approach is applied, operationalised or used across different contexts or situations. Drawing upon the metaphor in Figure 2, this symbolises how the magnifying glass is moved or applied. Intentional practice as a set of methods is currently operationalised in three ways: (1) models, (2) process steps and (3) critical questions.

#### 4.6.1. Models

Models provide a method to guide the operationalisation of wellbeing solutions and make the “approach” practical for context. Models conceptually organise the relationship between outcomes (“what”) and “how” this is actioned through locally contextualised responses, strategies and intervention components.

##### 4.6.1.1. Life buoyancy model (LBM): growth-focused model of intentional practice

This is the foundational model underpinning intentional practice (described in detail: 10, 11, 30). It is framed and categorised as a program logic model, which includes a hierarchy of short-, medium- and long-term outcomes (“what”), with associated intervention components (“how”) and process-based features that operationalise how growth outcomes are delivered. This model supports the deconstruction of interventions into “common elements” or “core components”. This modular or common elements approach seeks to identify the “main ingredients” of an intervention (59), and has been drawn from both the therapeutic (59, 60) and the program design and implementation literatures (61, 62). The LBM model is designed to be populated with contextualised content, and provides a method to articulate an intervention's “theory of change or growth”. This model has been adapted from established implementation science logic modelling (see 11, 30).

##### 4.6.1.2. Building block model

This model graphically operationalises “growth intent” through a building-block metaphor and the descriptors of: (1) awareness, (2) skills and (3) mindsets. These descriptors deconstruct larger growth outcomes (e.g., resilience), social-emotional skills (e.g., mindfulness) or goals (e.g., strengthened wellbeing) into “building blocks of growth intent”. It supports a scaffolded and targeted approach to the design and delivery of contextualised wellbeing solutions (see 49).

##### 4.6.1.3. Other models

There are other models that operationalise intentional practice to context. This includes two key models (What-What-How®, Activation Cycle) that translate intentional practice within moment-to-moment supporting relationship (e.g., intentional caregiving, teaching or support) and shared growth planning. These models are embedded within a competency-based training program (63).

#### 4.6.2. Process steps

Intentional practice can be operationalised through process-based steps to guide the design, adaptation and implementation of wellbeing solutions across different contexts. These process steps draw upon both the approach and key models (e.g., LBM). Process based steps and case examples are provided in the literature as follows:

• Trauma-informed care environments (30).
• The design and implementation of complex or multi-component wellbeing and resilience programs (10).
• The personalisation of social-emotional learning programs across educational settings (11).
• The integration of strategies and components to build wellbeing and environmental outcomes (64).
• Case planning and formulation within counselling, coaching and clinical interventions (12).

#### 4.6.3. Critical questions

Intentional practice can be applied in both highly simple and complex ways. In its simplest manner, intentional practice can be readily operationalised as “critical questions” that are brought to the design, review and implementation of existing or new wellbeing solutions. Table 4 identifies a selection of critical questions that can be wrapped around the design and implementation of: (1) moment-to-moment support, (2) evidence-based programs and (3) whole-of-community interventions (e.g., practice framework). These critical questions are designed to “ground” the wellbeing solution in its design, adaptation and implementation. In its most simplest manner, intentional practice can be operationalised simply as: “what is the intent of this intervention or wellbeing solution?” and “how am I actioning this intent?”.

**Table 4:** Critical questions underpinning the design, adaptation and implementation of wellbeing solutions.

Intentional support (moment)	Evidence-based program (EBP)	Whole-of-community (system)^a^
What is my intent right now?	What is the intent of this EBP?	What is the intent of this whole-of-community approach (e.g., wellbeing strategy, therapeutic framework)?
What outcomes am I working towards?	What are the intended outcomes of this EBP?	What are the outcomes we want to achieve?
How am I working to achieve these outcomes?	What are the core components or active ingredients of this EBP?	What are the core components that are central to the delivery of our whole-of-community approach?
What energy am I bringing to this supporting role?	What energy or intent am I bringing to this EBP?	What energy or intent underpins our whole-of-community approach?
Am I being “growth-focused” or bringing a “growth intent”?	Am I holding onto a “growth-focused” intent in the delivery of this EBP?	To what degree is “growth” as an approach, energy or intent visible in our service delivery?
Is my support having an activating or growth effect?	Is this EBP delivering growth outcomes?	Are our services and responses having an activating or growth effect?
Is it possible I could be causing unintentional harm (suppressing effects)?	In what ways could this EBP cause unintentional harm (suppressing effects)?	Is it possible that any of our services, responses and practice approaches are causing unintentional harm (suppressing effects)?

^a^ Adapted from Raymond (30).

## 5. Applications

This section is dedicated to summarising the purported applications of intentional practice across real-world settings. No conclusions regarding the relative effectiveness (or lack of effectiveness) of intentional practice is formally offered. The section moves from generalised to contextualised applications.

### 5.1. Generalised applications: complementary vs. standalone

Intentional practice can be applied to support the design, adaptation and implementation of wellbeing solutions in either a “complementary” or “standalone” manner. When it is applied in a complementary manner, intentional practice is designed to be an adjunct to other implementation or knowledge translation models. In this complementary application, individual features of intentional practice are applied at the discretion of the knowledge user or community. For example, individual (or collective) intentional practice features such as language (Table 2), principles (Table 3), approach, critical questions (Table 4) or models (e.g., LBM) can be overlaid existing intervention planning processes.

In contrast, when intentional practice is applied in a standalone manner, then the design, adaptation or implementation process is founded upon the following features:

• Intentional practice principles and language.
• Ongoing attention being paid to the lens or approach of mindfulness, intentionality and growth.
• Critical questions.
• A clear logic or intent between the outcomes (“what”) and the “how” and this is documented in a logic model or framework (e.g., Life Buoyancy Model, What-What-How® or other implementation science logic models).

The following section details a range of contextual applications of intentional practice, which draws more heavily upon its complementary functions.

### 5.2. Clinical, teaching and caregiving roles

Intentional practice asks supporting adults (e.g., caregivers, teachers, counsellors, practitioners, coaches, clinicians) to bring a lens of mindfulness, intentionality and growth to their support, and their moment-to-moment interactions. It uplifts the potential of every interaction being an opportunity for growth and learning within the support and care provided to others. In application, this can be framed as “intentional caregiving” or “intentional teaching”, or the formal role descriptor of “intentional practitioner”. In its simplest application, it can be operationalised as a supporting adult asking the critical question: “what is my intent right now in the care and support provided?”. For a clinician or practitioner, critical questions might include: “what outcomes am I working towards with my client during this contact?” and “how will I act to support the delivery of these outcomes?”. In other words, the practitioner brings ongoing mindful awareness to the “what” and the “how” within the design and delivery of the contextualised intervention, or the moment-to-moment support and care provided to another.

Intentional practice, as operationalised as a set of methods through the LBM, offers a case formulation framework to design (and co-design with a client) a clinical or non-clinical intervention or support plan (12). It supports the design of contextualised interventions similar in function to the “case formulation process” associated with cognitive behavioural therapy (40). The modelling provides a method for clinical and non-clinical personnel to work together and develop a “shared intent” in the design and formulation of a contextualised mental health and wellbeing solution. This may offer utility in “task sharing” contexts where non-clinicians (e.g., support workers, case managers) are being increasingly asked to be active in the planning and implementation of mental health and wellbeing interventions. As the language of “intent” is not linked to a specific discipline, it offers a common language for multi-disciplinary teams (e.g., doctors, nurses, social workers, support workers) to co-construct contextualised interventions. In this shared planning, there may still be key roles for content experts or knowledge brokers (e.g., psychologist, psychiatrist) to lead the knowledge transfer process (65).

### 5.3. Strengthening the implementation of existing evidence-based programs

Intentional practice was conceptualised to support the design and delivery of safe and higher impact wellbeing solutions (or interventions). The language, approach and methods can be overlaid existing interventions (in an umbrella fashion). In its most simplest manner, it can be operationalised as critical questions such as: “what is the intent of this intervention?”, “what outcomes are we working towards?”, and “how are we actioning this intent?”. These critical questions are designed to be embedded within agency or team reflective and communication processes, and when this occurs, it is postulated this will strengthen the delivery of existing evidence-based programs.

Through its logic modelling framework (LBM), intentional practice provides a method to deconstruct existing evidence-based interventions, where the relationship between the outcomes (“what”) and the core intervention components (or “how”) are named and articulated. This provides an opportunity to name the intent of individual evidence-based programs, and describe how they position and integrate alongside other interventions, where they exist. For example, the intent and associated outcomes of a program designed to manage aggressive client behaviour vs. an intervention designed to build social-emotional skill capacity (see 30).

### 5.4. Culturally responsive wellbeing solution design and implementation

Cultural determinants are a key intervention design consideration. Within an Australian context, there are significant socio-cultural, institutional and historical factors that impact on Aboriginal people, which must be uplifted into the design, adaptation and implementation of wellbeing solutions (66). Culturally responsive interventions uphold the principles of empowerment, co-design, self-determination and validating existing knowledge systems in the design and implementation of responses, care and programs impacting on Aboriginal people (see 67). The underpinning approach and principles of intentional practice, for example: “embrace complexity and diversity”, “side-by-side co-creation”, and “integrate wisdom and evidence” (see Table 3), share alignment with culturally responsive design principles. It is therefore postulated that intentional practice may offer a culturally responsive approach and set of methods to capture and magnify the knowledge systems of Aboriginal people in the design and implementation of Aboriginal led wellbeing solutions. In an early case example, Raymond and Lappin (68) describe the development of a contextualised wilderness-adventure program for young people with offending backgrounds, with very high Aboriginal representation. The program integrated best-practice trauma-informed principles with local cultural knowledge systems, as documented through the LBM logic model, and underpinned by the shared language of “intentionality”.

### 5.5. Contextualised delivery of trauma-informed practice

Trauma-informed practice is characterised by a set of best-practice principles that are actioned through care, support and teaching processes (20). There is a lack of consistent literature guidance in terms of how trauma-informed practice can be operationalised by systems, agencies and individuals to respond to the contextualised needs of people with trauma-based backgrounds. Intentional practice offers some unique methods and insights here (30).

Drawing upon the foundational principles in Table 3, intentional practice upholds the importance of: (1) individual understandings of trauma impact, (2) developing personalised wellbeing solutions that integrate best-practice trauma-informed principles with local knowledge systems, and (3) the co-creation of trauma-informed responses and interventions, such that a shared intent for healing and growth is articulated within a support community. As noted by Raymond (30), this can be practically operationalised through the design and implementation of whole-of-program trauma-informed frameworks (or therapeutic practice approaches), adaptation of evidence-based trauma interventions, and ultimately through moment-to-moment care and support. The latter includes the delivery of personalised responses (or micro interventions) that individually responds to the needs and context of the person being supported, in any moment of time.

### 5.6. Contextualised wellbeing solutions with fidelity

The paper has highlighted the importance of evidence-based interventions being adapted to context. Adaptation should occur in an intentional and evidence-based manner (35), which requires balancing both “flexibility” (or contextualisation) and “fidelity” within intervention design and implementation (69). Intentional practice is postulated to offer utility in this regard. This is explained as follows.

Intentional practice is “process” driven. It is focused on intentional program design by “describing” the process to design and implement wellbeing solutions, as opposed to “prescribing” specific interventions or programs. In other words, it brings fidelity to process, where wellbeing solutions are developed and adapted through a lens of high awareness, growth and intentionality, and integrating best-practice science into intervention design through a logic model framework and co-construction (wherever possible). In other words, it offers a “flexible method for program developers to bring creative flair to their work, but within a framework of intentionality and awareness of intent, desired outcomes (“what”) and method (“how”)” (10, p. 52). When a contextualised wellbeing solution is documented through the LBM logic model, the opportunity exists for “contextualised fidelity” (quality delivery) to be operationalised and assessed against the documented logic model (for further discussion see 10).

### 5.7. Complex programming and community capacity building

Intentional practice has been applied to support the design and implementation of complex programming. These are programs that include multiple intervention components, are delivered across multiple layers (individual, workgroup/classroom and organisation/school) or agency sites, and include cohorts with heterogeneous or complex needs (e.g., trauma). There are published examples of intentional practice informing the design and implementation of these programs across positive psychology (10), wilderness programming (68) and trauma-based residential care settings (30). Other applications include across positive education, community services and community-based mental health.

A feature of complex programs is that wellbeing solutions (or evidence-based programs) are nested within wellbeing solutions. This is representative of a community capacity building program targeting mental health and wellbeing outcomes. The community may be a school, agency or geographic area. A complex program of this type can be operationalised through a whole-of-community wellbeing framework, an evidence-based social-emotional learning (SEL) or wellbeing program, and moment-to-moment trauma-informed practice. Intentional practice offers a common language, approach and set of methods that spans the design, adaptation and implementation of all nested wellbeing solutions.

A literature example of a nested complex program is Resilient Futures (10, 49). This was a multi-site delivery of a wellbeing and mental health program which included the contextualised delivery of wellbeing and resilience skills to over 1,400 young people from disadvantaged backgrounds and agencies spanning alternative education, health, child welfare and mental health. Intentional practice operationalised the: (1) program logic model framework (10), (2) delivery of localised interventions for wellbeing and resilience skill development, and (3) delivery of intentional case management and trauma-informed coaching support. The Resilient Futures' case example provided preliminary evidence that intentional practice was highly translatable and practical across multi-disciplinary settings, and a central driver of the program's purported success (49).

### 5.8. Cross-discipline integration

Across diverse literatures, there is increasing call for approaches that support conceptual, methodology and functional integration of scientific disciplines (70, 71). Intentional practice responds to this need by offering a non-discipline aligned language, approach and set of methods.

An example of intentional practice's potential to foster inter-disciplinary integration is demonstrated through a case-study wellbeing solution that brought together the wellbeing (positive psychology) and sustainability literatures. Raymond and Raymond (64) conceptualised a program designed to increase landowner tree planting, as well as promote higher levels of subjective wellbeing. Intentional practice, as operationalised through the LBM logic modelling, brought together outcomes and intervention components drawn from both disciplines. This included: (1) mindfulness training in nature, (2) nature exposure, (3) education, (4) value clarification processes and (5) environmentally driven call-to-action activities.

## 6. Discussion

Reducing the “science-to-practice” gap in the delivery of clinical, trauma-informed and growth-focused services (or wellbeing solutions) has attracted increasing interest across multi-disciplinary settings. The accumulation of new scientific knowledge within the implementation science literature remains slow (4), and this paper has sought to introduce and inspire novel insights that tap the interface between theory and application, a key area of development for the field (5). To this effect, this paper offers the following higher-level insights.

First, the paper offers broader understandings of the multi-levelled nature of interventions, as operationalised from the system, programmatic and moment-to-moment levels. This represents a more granular and nuanced construction of wellbeing solution design and implementation than is traditionally seen in the implementation science literature. The moment-to-moment level, a key feature for implementing trauma-informed practice (30), highlights the role of individual cognitive activities (or the intent of a practitioner, teacher, caregiver etc) in the process of intervention design and implementation (in the moment). It brings awareness to the interface between “intent” and “actions”, and associated decision making processes.

Second, the paper offers further support for the integration of the complexity and implementation science literatures (6, 7), and the role of “context” to understand the degree best-practice science becomes “sticky” in application. It upholds the importance of fidelity of “process” in the design, adaptation and implementation of interventions (or wellbeing solutions). This includes a focus on “describing” key methods to develop wellbeing solutions, as opposed to “prescribing” an individual intervention. For this reason, drawing upon the implementation model categories proposed by Nilsen (5), intentional practice is most closely aligned to the “process model” category.

The paper also supports the importance of iterative and dynamic planning processes within knowledge translation (15). The argument is made that the embracement of complexity within implementation science will require people, programs and communities to come together in “shared intent”, through iterative and participatory communication, to design and implement contextualised interventions (8). The bringing together of people is the hallmark of best-practice knowledge translation, and this paper has introduced the possible role of common language and approaches (founded upon intentionality) to aide this process.

Fourth, the paper has highlighted the importance of deconstructing scientific knowledge in practical terms (13), such that it is accessible for non-scientific knowledge users. It has introduced the cognitive short-cuts of “stickiness” (or “sticky”) and “intentionality” (or “intent”) to operationalise the outcomes and processes of implementation science, respectfully. These examples invite implementation scientists to consider what other key concepts can be deconstructed and made sticky in action for both scientific and non-scientific audiences.

At the most practical level, the paper has introduced “intentional practice” as a common language, approach and set of methods that is purported to support the design, adaptation and implementation of wellbeing solutions (from the “system” to the “moment”). Developed from the implementation science literature, it has been iterated and refined through participatory processes, which remains ongoing today. Intentional practice as an “approach” asks knowledge users to hold onto principles of complexity and contextuality, and bring a lens of mindful awareness, growth and intentionality to everything they do. As a set of methods (“methodology”), it offers models, critical questions and process-based steps to design and implement wellbeing solutions that integrate the science of wellbeing, resilience, growth and trauma-informed practice (etc) with local knowledge systems and existing interventions. In application, the features (e.g., language, principles, approach or methods) can be applied either in a standalone or complementary manner. Intentional practice does not seek to replace existing knowledge systems or implementation frameworks, but instead, offers a way to contextualise and strengthen the implementation of existing tools, approaches and evidence-based programs in a manner that is postulated to deliver safer and higher impact outcomes.

The paper has highlighted the potential value for both scientists and non-scientists in having a shared language, approach and set of methods. Of most importance, it aides both collaboration and “task sharing” (41). Across mental health settings, non-clinicians (e.g., support workers, case managers) are being increasingly asked to be active in the planning and implementation of mental health and wellbeing services. Having a shared language and set of methods (e.g., critical questions) between clinicians and support workers can aide rapid information sharing, knowledge translation, and intervention planning and design processes. Intentional practice may offer utility for content experts (65) and purveyor organisations (38) in knowledge translation activities. These brokers and purveyors may draw upon intentional practice language, approaches and critical questions (singularly or collectively) to organise the design and articulation of wellbeing solutions, as co-constructed with the members of the community they are supporting.

Intentional practice can be trained to caregivers, teachers, practitioners, coaches, clinicians, program developers and researchers. There has been a call for wider dissemination and training of implementation science constructs (72), and the inclusive language and processes of intentional practice may offer utility in this regard. Preliminary research provides optimism that the language, approach and methods are both sticky and practical for non-scientist knowledge users, and can significantly aide the design, adaptation and implementation of contextualised wellbeing solutions (49). However, this paper makes no conclusions regarding its relative effectiveness or value, alongside existing implementation science models. Intentional practice would appear to offer most utility as a process model to complement (rather than replace) existing implementation science and knowledge translation frameworks. Further empirical analysis is required with consideration to the following priority questions:

• To what degree is the language of intentional practice “sticky” for multi-disciplinary scientific and non-scientific knowledge users?
• How do diverse knowledge users construct and apply intentional practice?
• To what degree can intentional practice become a common language, approach and set of methods across systems, programs and communities?
• What are the barriers and facilitators of embedding intentional practice across systems and communities?
• What is the complementary value of intentional practice alongside other implementation models, approaches and theories?

## 7. Summary

The move towards valuing complexity and contextualisation in the design and implementation of wellbeing solutions will require innovative models and approaches that empower people, programs and communities to come together in “shared intent”. In other words, to have a shared understanding of the intervention context, what is the intent of the wellbeing solution and its implementation, and how this can be collectively actioned and delivered. This paper points to the potential role of common language and approaches that build a bridge between science and practice, and scientists and non-scientists alike, to deliver this outcome.

## Data Availability

The original contributions presented in the study are included in the article, further inquiries can be directed to the corresponding author.
